# Some Hints on the Most Efficient Modes of Administering Medicines

**Published:** 1845-03

**Authors:** 


*Some Hints on the most Efficient Modes of Administering Medicines.* By a practitioner of half a century. — Many of the most important discoveries and improvements in medical science are rendered comparatively useless, in consequence of being unskilfully applied to actual practice. In no department of knowledge is this defect more conspicuous than in therapeutics. Man (and I believe the same remark applies to all created beings) is born with a kind of instinctive antipathy to physic, which antipathy he retains from the cradle to the grave. Look at the ingenious spoons that have been invented to force physic down the throats of infants! Observe the mantel-pieces of sick chambers, and count how many phials are either uncorked, or only half emptied! How great a proportion of mankind hate the very name of physic! If the stomach is apt to turn at the thought of medicine, when we are in health, how much less capable is it to bear nauseous drugs in the various forms of disease, nine-tenths of which affect the stomach sympathetically with squeamishness, nausea, and aversion to food as well as physic? The evil consequences of nauseous forms of medicine being used in sickness, are great beyond all calculation or belief. One result is, that medicine is not taken in sufficient quantity, sufficiently often, or for a proper length of time.

What practitioner will fail to recognise the following picture of almost daily occurrence? A medical man is in anxious attendance on a patient — say a lady after confinement, and threatened with some grave malady, peritonitis, for instance—he prescribes what he conceives to be active and efficient remedies for the night, and gives strict injunctions to the nurse. In the morning, when he calls, he meets the nurse on the stairs. Have you given your mistress the medicines punctually? Most punctually, sir. Well, what has been the effect? "Brought everyting up again sir." What, all? "Every drop, sir—and I thought she would have brought her very heart up with it." After such intelligence, the feelings of the doctor, on entering the chamber, are not particularly enviable. Now all this is more frequently owing to the form than the substance of the medicine exhibited.

In chronic diseases, where the remedial process is necessarily chronic also, we are daily baffled by the repugnance, nay, the resistance of the patient to a protracted course of physic. Yet it might very generally be so contrived, that the patient would desire rather than loathe his medicines.

I am aware that in some acute diseases, the state of nausea itself is desirable and salutary. But it is not the mere nausea or sickness which lessens the velocity of the circulation, opens the secretory vessels, and checks inflammation. These remedial processes depend much upon the *quantity* of medicine, say antimony, which the patient can bear in order to induce them. Thus double or triple the quantity of tartrate of antimony will be borne, before sickness is induced, if given in an effervescing draught, as compared with the same medicine given in plain water. And the remedial effects will be in proportion. This is a truth that should ever be held in mind, and the principle was well understood by Rasori, Thomasini, and others. The contra-stimulant effects of antimony are trifling during the nausea and sickness at the beginning. It is when the *tolerance* is acquired that the inflammation or high fever is controlled.

But there is a large class of diseases in which the stomach is morbidly irritable, and where nauseating medicines are positively injurious. Putting aside the multitudinous forms of dyspepsia, we have affections of the uterus, the kidney, the liver, the pancreas, &c., where the stomach is prone to disordered function, and where it is of the greatest consequence to exhibit medicines in forms that will tranquillize rather than nauseate the stomach. Diseases and disorders of the kidney are now acknowledged to be much more frequent than they were formerly suspected to be—and these are very generally attended with gastric irritability. In these it is of great importance not to ruffle the stomach by medicines. In affections of the brain, now so exceedingly common in consequence of the advanced stage of civilization, and the operation of various perturbing moral causes, the stomach is often the organ most conspicuously deranged—and we are not seldom foiled in the exhibition and perseverance of proper remedies, from the sympathetic disorder of stomach.

Nine-tenths of the cures that are said to be performed by homœopathy, result from the spare diet and the nullity, as it were, of medicine employed. Of all the medicines that are prescribed by the physician, the class of salines are the most generally beneficial, as opening the secretory organs, as the skin, the liver, the kidneys, &c., besides improving the state of the blood, and restraining febrile action in the constitution. These, when exhibited in an effervescing state, are far more palatable, as well as more efficacious, than when given in a plain form.

Tonics, on which the routine practitioner so much relies, and which he exhibits with no sparing hand, are more frequently injurious than beneficial. They give a feeling of tone for a time; but they lock up the secretions, increase too much the appetite, and lay the foundation for future states of plethora, congestion, or indigestion.

Now saline effervescents may be made the vehicle for many of the most powerful tonics, and indeed the most potent medicines which we possess. The citrate of iron, colchicum, antimony, arsenic, quinine, iodine, &c., &c., may all be exhibited in a form that increases their remedial efficiency, and lessens their tendency to nauseate the stomach.

*Bulletin of Medical Science.*

